# Effects of Strigolactone on *Torreya grandis* Gene Expression and Soil Microbial Community Structure Under Simulated Nitrogen Deposition

**DOI:** 10.3389/fpls.2022.908129

**Published:** 2022-06-02

**Authors:** Chenliang Yu, Qi Wang, Shouke Zhang, Hao Zeng, Weijie Chen, Wenchao Chen, Heqiang Lou, Weiwu Yu, Jiasheng Wu

**Affiliations:** ^1^State Key Laboratory of Subtropical Silviculture, Zhejiang A&F University, Hangzhou, China; ^2^School of Forestry and Biotechnology, Zhejiang A&F University, Hangzhou, China; ^3^NFGA Engineering Research Center for Torreya grandis ‘Merrillii’, Zhejiang A&F University, Hangzhou, China

**Keywords:** microbial community, nitrogen deposition, RNA-seq, strigolactone, *Torreya grandis*

## Abstract

Nitrogen enters the terrestrial ecosystem through deposition. High nitrogen levels can affect physical and chemical properties of soil and inhibit normal growth and reproduction of forest plants. Nitrogen modulates the composition of soil microorganisms. Strigolactones inhibits plant branching, promotes root growth, nutrient absorption, and promotes arbuscular fungal mycelia branching. Plants are subjected to increasing atmospheric nitrogen deposition. Therefore, it is imperative to explore the relationship between strigolactone and nitrogen deposition of plants and abundance of soil microorganisms. In the present study, the effects of strigolactone on genetic responses and soil microorganisms of *Torreya grandis*, under simulated nitrogen deposition were explored using high-throughput sequencing techniques. *T. grandis* is a subtropical economic tree species in China. A total of 4,008 differentially expressed genes were identified in additional N deposition and GR24 treatment. These genes were associated with multiple GO terms and metabolic pathways. GO enrichment analysis showed that several DEGs were associated with enrichment of the transporter activity term. Both additional nitrogen deposition and GR24 treatment modulated the content of nutrient elements. The content of K reduced in leaves after additional N deposition treatment. The content of P increased in leaves after GR24 treatment. A total of 20 families and 29 DEGs associated with transporters were identified. These transporters may be regulated by transcription factors. A total of 1,402,819 clean reads and 1,778 amplicon sequence variants (ASVs) were generated through Bacterial 16S rRNA sequencing. Random forest classification revealed that Legionella, Lacunisphaera, Klebsiella, Bryobacter, and Janthinobacterium were significantly enriched in the soil in the additional N deposition group and the GR24 treatment group. Co-occurrence network analysis showed significant differences in composition of soil microbial community under different treatments. These results indicate a relationship between N deposition and strigolactones effect. The results provide new insights on the role of strigolactones in plants and composition of soil microorganisms under nitrogen deposition.

## Introduction

Atmospheric nitrogen deposition has increased more than 10 times in the past 150 years and is expected to double by 2050, due to combustion of fossil fuels and wide use of agricultural fertilizers (Galloway and Cowling, 2002). Nitrogen deposition significantly affects the balance of various ecosystems under global environmental changes (Lin et al., 2020). In addition, nitrogen deposition plays an important role in global nitrogen cycle (Xu et al., 2019). China is among the top three highest nitrogen deposition regions in the world owing to rapid economic development in the country (Holland et al., 1999; Dentener et al., 2006; Wang et al., 2011; Lin et al., 2020). Nitrogen deposition rates was 30–70 kg/ (ha*yr) in China (Liu et al., 2013; Ti et al., 2021; Wu et al., 2021; ha*yr) Studies predict that these regions will exhibit the highest atmospheric nitrogen deposition in the world in future (Wang et al., 2011). A moderate amount of atmospheric nitrogen deposition promotes plant growth. However, when the ecosystem nitrogen reaches saturation, continuous increase in nitrogen deposition negatively affects plant growth (Lu et al., 2014). Excessive nitrogen levels as a results of nitrogen deposition causes phosphorus deficiency in soils, which in turn changes the physical and chemical properties of soil and reduces plant productivity (Lü and Tian, 2007). In addition, nitrogen deposition reduces soil pH, reduces plant diversity, and affects composition and activity of microbial communities (Clark and Tilman, 2008; Lu et al., 2014). Moreover, high levels of nitrogen affects decomposition of litter by microorganisms and mineralization of organic matter, ultimately changing the content of soil nutrient elements (Elser et al., 2007; Dima et al., 2015).

Soil microorganisms play a key role nutrient cycling in forest ecosystems (Dom et al., 2021). The research on soil microorganisms under the nitrogen deposition mainly focuses on the changes of microbial community structure in different forest types or different ecosystems (Wang et al., 2018a,b). Previous findings show that long-term nitrogen deposition has adverse effects on soil microorganisms (Díaz-Álvarez et al., 2018). Atmospheric nitrogen deposition can directly or indirectly affect growth, reproduction, and activity of forest soil microorganisms (Berg et al., 2011). Moreover, it can change the number, community structure, and function of soil microorganisms (Treseder, 2010). These changes ultimately affect material transformation and nutrient availability in soil (Treseder, 2010; Cusack et al., 2011). Previous studies report that excessive nitrogen deposition leads to decrease in fungal biomass in soil, changes fungal bacterial biomass ratio, affect diversity of ectomycorrhizal fungi species. Furthermore, high nitrogen deposition level is associated with decrease in soil enzyme activity and respiration rate, and change in microbial substrate utilization mode (Waldrop et al., 2004).

Strigolactones (SLs) are plant hormones produced in carotenoid biosynthesis pathway (Matusova et al., 2005). Studies report that SLs inhibit growth of plant lateral branches, induces seed germination, and stimulates hyphal branching of arbuscular mycorrhizal fungi (Kapulnik and Koltai, 2014; Al-Babili and Bouwmeester, 2015; Shindo et al., 2020). Secretion of strigolactones by roots plays an important role in fungus and host before mycorrhizal colonization (Müller et al., 2019). Arbuscular mycorrhizal fungi increase the absorption area of host plant roots, release organic acids and soil enzymes, activate soil phosphorus, and enhance the host phosphorus absorption capacity (Huang et al., 2018; Higo et al., 2020). Many studies have shown SLs has the potential ability to regulate plant roots and rhizosphere microorganisms interaction (van Zeijl et al., 2015; Carvalhais et al., 2019). There are clear evidence present that SLs promote biotic stress resistance against specific bacterial and fungal phytopathogens (Marzec, 2016). SLs are implicated in plant nutrient absorption. Nutrient deficiency induces SLs synthesis resulting in plant developmental plasticity (Pandey et al., 2016). Increase in level of SLs under a low-phosphorus environment is correlated with inhibition of aboveground branches and stimulation of lateral root growth (Brewer et al., 2013). Low nitrogen level promotes increase in the synthesis of SLs (Yoneyama et al., 2012; Shindo et al. 2020). Differences in biosynthesis or transport of SLs can affect development of shoots in response to supply of N elements (Cochetel et al., 2018). More than 25 natural strigolactones have been isolated from plant roots (Oancea et al., 2017). The synthetic strigolactone analog GR24 is widely used to explore the biological role of SLs (Umehara et al., 2008).

*Torreya grandis* “Merrillii” is a gymnosperm, and a member of the *Torreya* genus and Taxaceae family (Gao et al., 2021). It is a species of *Torreya grandis* Fort.ex Lind. that has undergone asexual reproduction (Li and Dai, 2007). *T. grandis* is a multi-purpose excellent economic tree used for production of fruit, oil, medicine, wood, greening, and ornamental purposes (Li and Dai, 2007; Chen et al., 2020). *T. grandis* mainly grows in Zhejiang Province in China. The purpose of this study was to explore whether SLs can improve stress response in *T. grandis* under additional N supply. In the present study, the effects of SLs on gene responses and soil microorganisms of associated with *T. grandis* subjected to simulated nitrogen deposition were evaluated using high-throughput sequencing techniques. The findings of the present study provide new insights and basis for further studies on the response of subtropical forest plants and soil microorganisms to nitrogen deposition.

## Materials and Methods

### Plant Materials and Treatments

One year old *T. grandis* plants were used in this study. The plants were treated with ammonium nitrate (NH_4_NO_3_) to simulate additional N deposition. The concentration of nitrogen used was 160 kg N ha ^−1^ yr. ^−1^. The amount of spraying per year was converted into the amount for each month. Strigolactone treatments comprising spraying of 10 μM of GR24 solution to the shoots of *T. grandis* seedlings. Spray with a small pot, 1 ml for each seedling. A total of 20 seedlings were used for each treatment. Samples were obtained for transcriptome sequencing after 24 h and 72 h of treatment. We treated them once every 1 month. Further, samples were collected for element content determination after 2 months of treatment. Soil samples were obtained for bacterial 16S sequencing after 2 months.

### Determination of Nutritional Element Content

The samples were subjected to concentrated nitric acid perchloric acid digestion and analysis of nutritional elements was performed using inductively coupled plasma mass spectrometry (ICP-MS; Xseries 2 ICP-MS, Thermo, United States). A weight of 0.1 g of the sample was transferred to a 100 ml conical flask, and 10 ml of concentrated nitric acid added. The conical flask was then placed on a heating plate at 80°C for 30 min, and then gradually heated until the brown-red gas at the bottle mouth disappeared. Further, 2.5 ml of perchloric acid was added; then, the mixture was heated to 180°C, heating was continued until the liquid became transparent. The mixture was cooled then diluted to a 50 ml volumetric flask with distilled water. Subsequently, the mixture was filtered into a 50 ml conical flask. The content of each element was then determined by atomic absorption spectrophotometer.

### Total RNA Extraction and Transcriptome Sequencing

TRNzol Universal Total RNA Extraction Reagent (DP424, TIANGEN Biotech, Beijing, China) was used to extract total RNA from the leaves of *T. grandis* subjected to different treatments. Nanodrop (Thermo, United States) was used to determine the purity (D260/D280) and concentration of RNA samples. Approximately 1 μg of RNA was obtained from each sample for library construction. mRNA with polyA tail was enriched by Oligo (dT) magnetic beads and randomly interrupted. One-stranded cDNA was synthesized with six-base random primers (random hexamers) by the M-MuLV reverse transcriptase system using mRNA as a template. Buffer, dNTPs, and DNA polymerase I were used to synthesize two-stranded cDNA. AMPure XP beads were used to purify the double-stranded cDNA. The purified double-stranded cDNA was then repaired, A-tailed, and connected to the sequencing adapter. Further, AMPure XP beads were used for fragment size selection. PCR amplification was conducted to obtain the final cDNA library. A total of 21 RNA-seq libraries were constructed. Sequencing was performed using the Illumina NovaSeq 6000 platform.

### Transcriptome Data Quality Control and Analysis

Adapter sequence was removed from the obtained raw data then reads with low quality and high unknown base content were filtered out to obtain high-quality clean reads. The clean reads were compared with previously assembled transcript sequence using hisat2 software. Count data were acquired using feature Counts software (Han et al., 2020). Transcripts per million (TPM) method was used to estimate gene expression using the Trinity software (Haas et al., 2013). DESeq2 R package was used to identify differentially expressed genes (DEGs) at a false discovery rate (FDR) < 0.05. GeneOntology (GO) and Kyoto Encyclopedia of Genes and Genomes (KEGG) enrichment analysis of DEGs was performed using clusterprofiler (3.4.4) tool, with correction of the gene length deviation. GO terms or KEGG pathways with corrected value of *p* less than 0.05 were considered significantly enriched by the DEGs.

### Weighted Gene Co-expressed Network Analysis

A total of 14,654 genes were obtained for further analysis based on the expression of more than 1 in 80% of the samples. Weighted gene co-expression network analysis (WGCNA) was conducted using WGCNA R package (Langfelder and Horvath, 2008). Hierarchical clustering on samples was performed using ward. D2 algorithm in hclust function. The soft threshold was calculated by picksoftthreshold function after ensuring that there was no outlier sample. The weighted adjacency matrix was constructed, and the related gene modules were identified based on the hierarchical clustering of the dissimilarity measure of the topological overlap matrix (TOM). A local network containing ion transport transporters related DEGs was established using the results of module division. A transcriptional regulation network was generated using Cytoscape software.

### Soil DNA Extraction and Sequencing

Total DNA of soil samples was extracted using TIANamp Soil DNA Kit (Code:DP336, TianGen Biotech, Beijing, China). Concentration and purity of the soil were determined using 2000 spectrophotometer, and detection conducted using 1% agarose gel electrophoresis. Total microbial DNA of each soil sample was used as the template for PCR amplification with bacterial V3 ~ V4 region-specific primers 341F (5′-cctacggggcgwgcag-3′) and 806R (5′- ggactachvggtwtctaat-3′). The PCR product was recovered by agarose gel electrophoresis after detection of target fragments using AxyPrep PCR Clean-up Kit Recovery Kit. Truseq nano DNA LT library prep Kit (Illumina company) was used to construct a sequencing library based on the purified PCR product. DNA PCR-free sample preparation kit was used to quantify samples on qubit and qPCR fluorescence quantitative systems. Sequencing was performed using IlluminaMiSeq platform.

Cutadapt (V1.9.1) quality control software^1^ was used to evaluate the sequencing quality of all 16S sequences, which were further grouped according to the different primers used for the samples. Usearch V10 software overlap was used to splice Clean Reads of each sample. Further, length filtering of the spliced data was performed according to the length range of different regions. Final valid data (non-Chimeric Reads) were obtained by denoising and removing chimeric sequences using dadA2 method in QIIME2 software (Bolyen et al., 2019). ASV sequences were compared with corresponding database sequences in QIIME2 software. NCBI database annotations were used to obtain the corresponding taxonomic information of each ASV. Specific species composition of each sample at each taxonomic level was obtained according to classification of ASVs and taxonomic status identification results. QIIME2 software was used to calculate Alpha diversity index and Beta diversity index for each sample.

### Co-occurrence Network Analysis

A co-occurrence network was constructed with each treated sample. The main ecological clusters of strongly correlated ASVs were then determined. Paired Spearman correlations between ASVs were evaluated, and correlations with Spearman coefficient less than 0.60 and value of *p* greater than 0.01 were removed. The main modules in the network were visualized using Gephi tool.^2^

## Results

### Transcriptome Sequencing Quality Analysis

Raw data was processed before analysis to ensure high quality during data analysis. The clean data were obtained by removing the adapter, and reads containing indeterminate base information and low-quality reads (Supplementary Table S1). A total of 20 million clean reads were obtained for each sample, with Q20 greater than 98% and Q30 greater than 94%. The sequencing error rate was 0.02–0.03%. These results indicate that the sequencing quality was good and the sequences can be used for subsequent bioinformatics analysis. Overall sample principal component analysis (PCA) was performed to explore the expression levels of genes in all samples. The results showed that the samples had good repeatability (Supplementary Figure S1). Details on identified unigenes are presented in Supplementary Table S2.

### Analysis of DEGs Related to N Deposition and SLs Response

**Figure 1:**
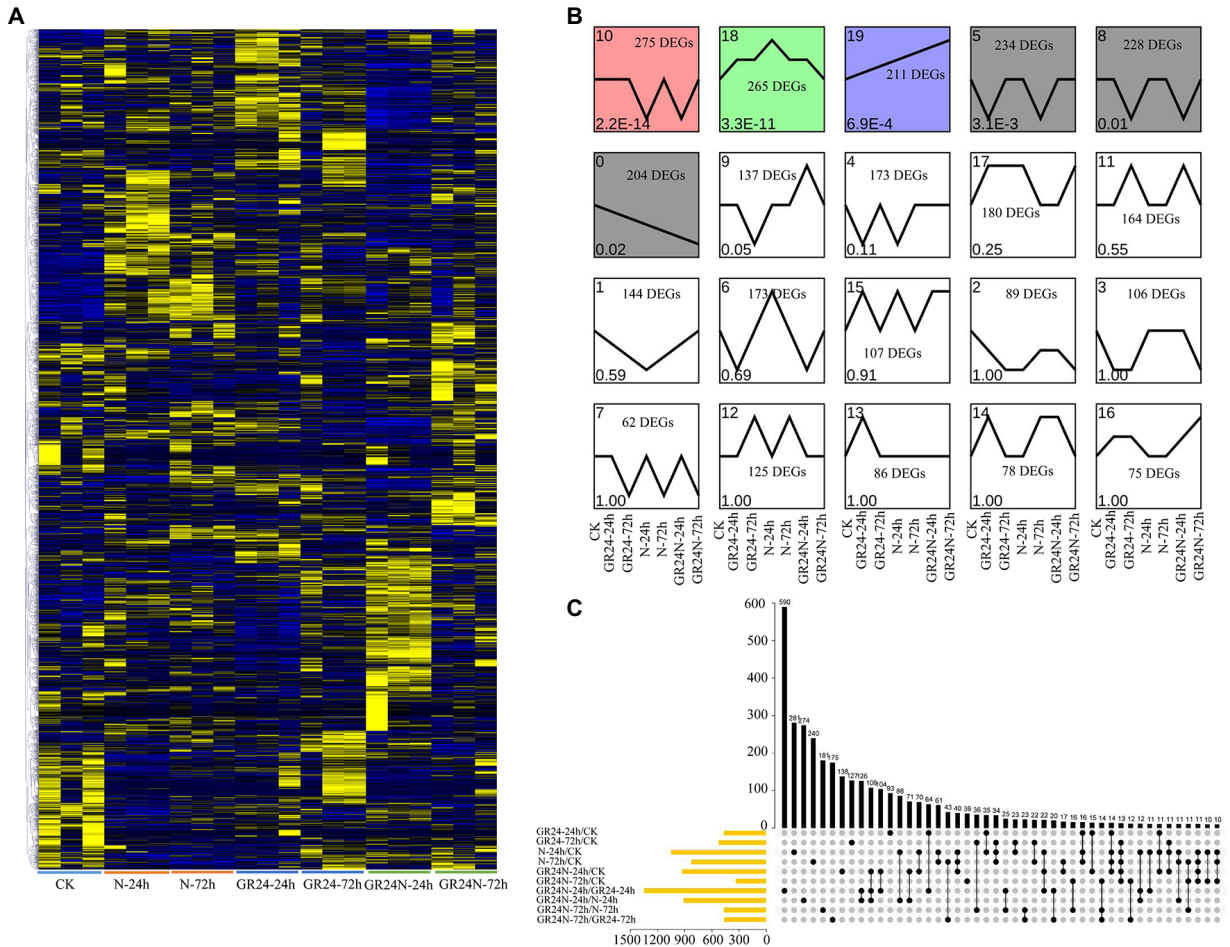
Identification of the DEGs between additional N deposition and GR24 treatments in Torreya grandis. **(A)** Expression profiles of the DEGs under additional N deposition or GR24 treatments were shown by a heatmap. The original expression values were normalized by Z-score normalization. **(B)** DEGs module expression trends by the line chart. The expression trend line graph of each sub-module, the horizontal axis is the sample, and the vertical axis is the average expression level of all the genes in the sample. The trend of value of *p* < 0.05 is significant. **(C)** Upset plot of the DEGs in different comparisons.

DESeq2 software was used to compare the expression levels of genes of *T. grandis* leaves under different treatments *p* < 0.05 was used as the screening criteria. DEGs for each comparison are shown in Supplementary Figure S2. Global DEGs profiles under additional N deposition or GR24 treatments were presented as a heatmap (Figure 1A). A total of 1,048 DEGs, including 556 upregulated and 492 downregulated genes, were identified under the additional N deposition treatment for 24 h. Further, 829 DEGs including 426 upregulated and 403 downregulated genes were identified for the 72 h treatment. A total of 468 DEGs, including 218 upregulated and 250 downregulated genes were identified after 24 h under GR24 treatment. A total of 527 DEGs, including 288 upregulated and 239 downregulated genes were identified after 72 h of GR24 treatment. A total of 931 and 335 DEGs were identified after 24 h and 72 h under additional N deposition+GR24 treatment (Supplementary Figure S2). A total of 4,008 non-redundant DEGs were obtained in all comparison groups (Supplementary Table S3). We also analyzed the expression trend of differentially expressed genes (Figure 1B). The results showed that there was significant difference in DEGs of cluster 0, 5, 8, 10, 18, and 19 (*p* < 0.05). Among them, the DEGs of cluster 19 increased in all treatments, while the expression of DEGs in cluster 0 decreased in all treatments. Notably, 281 and 240 DEGs were ascribed to treatment with additional N deposition for 24 h and 72 h, respectively (Figure 1C). In addition, 93 and 127 DEGs were attributed to GR24 treatment for 24 h and 72 h, respectively.

### Go and KEGG Enrichment Analysis of Differentially Expressed Genes

**Figure 2:**
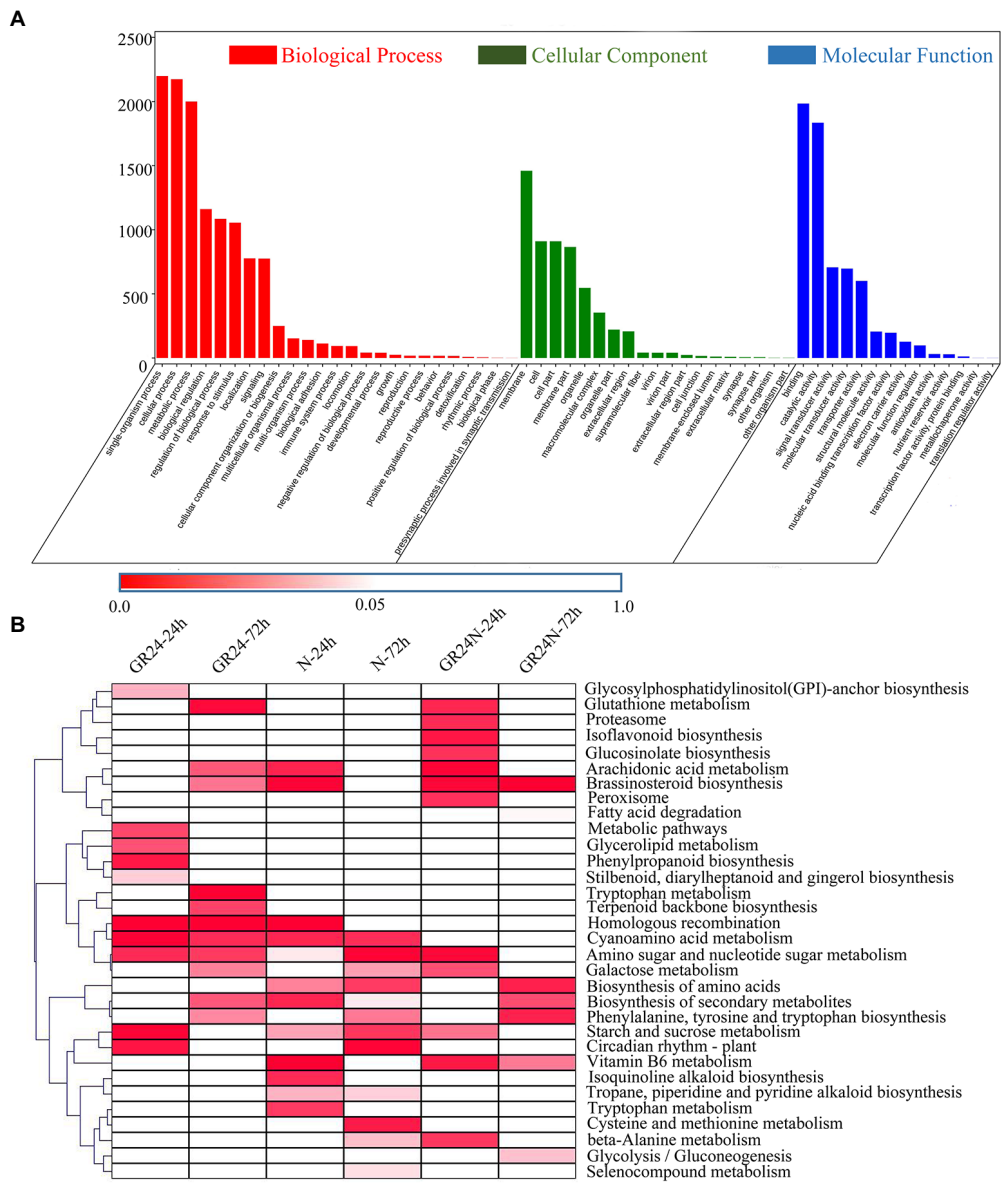
Enrichment analysis of the DEGs in different comparisons. **(A)** GO enrichment analysis of all the DEGs based on three GO terms: biological process, cellular component, and molecular function. **(B)** KEGG enrichment analysis of the DEGs in the six comparisons. The significant values of *p* of each KEGG term under different treatments were shown by a heatmap.

GO analysis of the DEGs was conducted to determine the main biological functions of these genes (Figure 2A). A total of 25 GO terms were significantly enriched in the biological process (BP) category. The top three significantly enriched terms in the BP category were single-organism process, cellular process, and metabolic process. The findings showed that 19 GO terms were significantly enriched in the cellular component with cell, cell part, and membrane part as the top three terms. A total of 14 terms were identified for molecular function category, with binding, catalytic activity, and signal transducer activity being the most enriched terms.

The DEGs were associated with enrichment of different KEGG metabolic pathways (Figure 2B). The results showed that 32 pathways were significantly enriched (*p* < 0.05). Arachidonic acid metabolism and Brassinosteroid biosynthesis were significantly enriched in the additional N deposition treatment group after 24 h and GR24 treatment group after 72 h treatment. Vitamin B6 metabolism was significantly enriched in the additional N deposition treatment group after 24 h and the additional N deposition+GR24 treatment group after 24-h and 72-h treatment. Phenylalanine, tyrosine, and tryptophan biosynthesis were significantly enriched in the additional N deposition treatment group after 72 h, GR24 treatment group after 72-h treatment, and additional N deposition+GR24 treatment group after 72-h treatment. Starch and sucrose metabolism and Circadian rhythm—plant were significantly enriched after 72-h treatment in the additional N deposition group and the GR24 group after treatment for 24 h. These results indicate that there was a crosstalk between N deposition and strigolactone.

### Nutritional Elements Analysis

**Figure 3:**
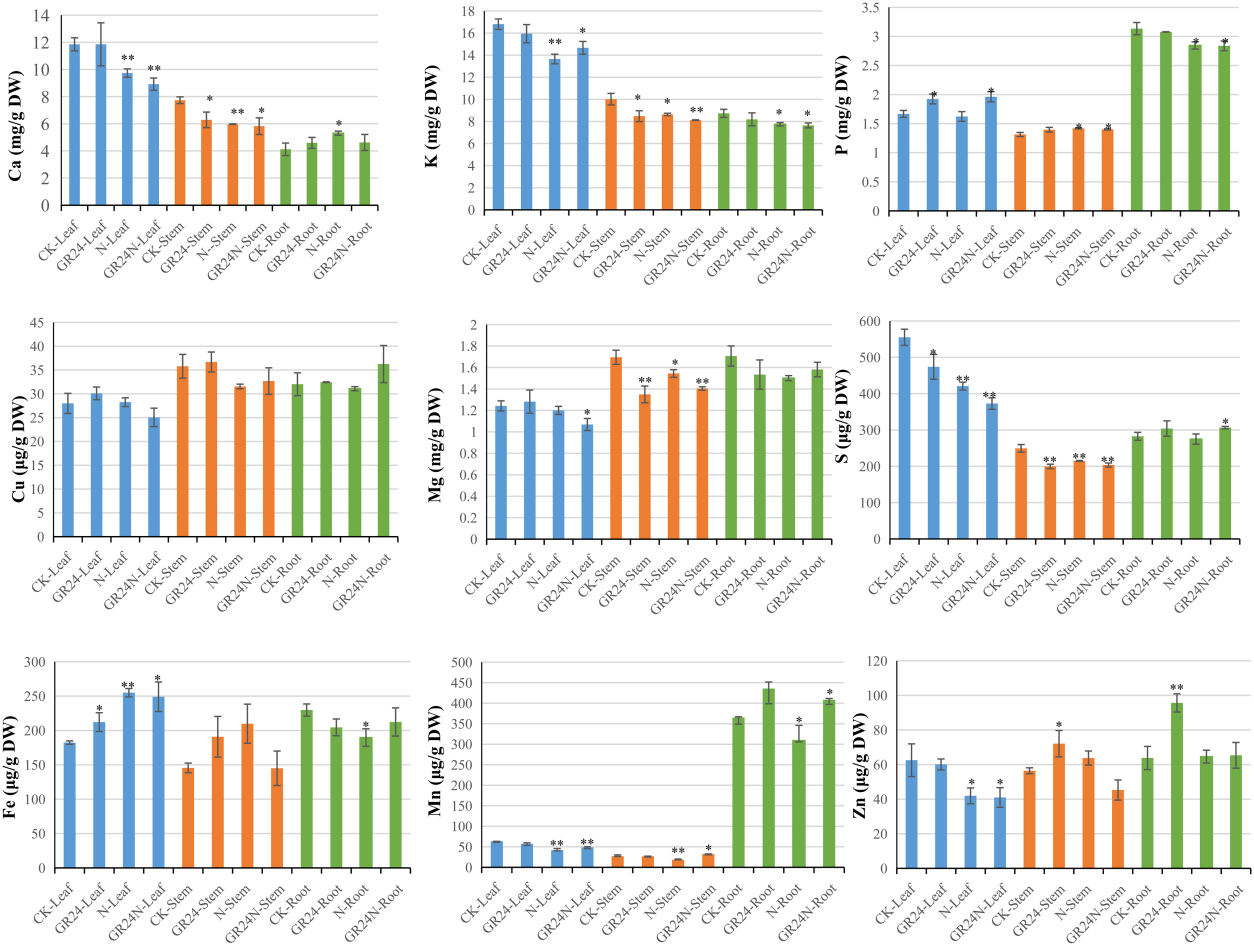
The characteristics of nutrient elements contents in *Torreya grandis* after additional N deposition or GR24 treatments for two mouths. “*” represent *p* ≤ 0.05, using unpaired student *t*-test; “**” represent *p* ≤ 0.01, using unpaired student *t*-test.

Increase in nitrogen deposition leads to decrease in soil inorganic phosphorus content and significant increase in soil N/P ratio (Blanes et al., 2013). Nitrogen deposition affects absorption of nutrients by plants. GO enrichment analysis showed that several DEGs were associated with enrichment of the transporter activity terms. Therefore, further analysis of common nutritional elements was performed (Figure 3). The results showed that additional N deposition treatment reduced the content of Ca in leaves and stems and increased the level of Ca in roots. The content of K reduced in leaves after additional N deposition treatment. The content of P increased in leaves after GR24 treatment. GR24 treatment reduced the content of Mg in stems. The three treatments reduced the content of S reduced in leaves. The content of Fe increased in leaves and reduced in roots after additional N or GR24 treatment. The content of Zn reduced in leaves after additional N treatment and increased in roots after GR24 treatment.

### Analysis of DEGs Related to Element Transporter Genes and Transcription Factors

**Figure 4:**
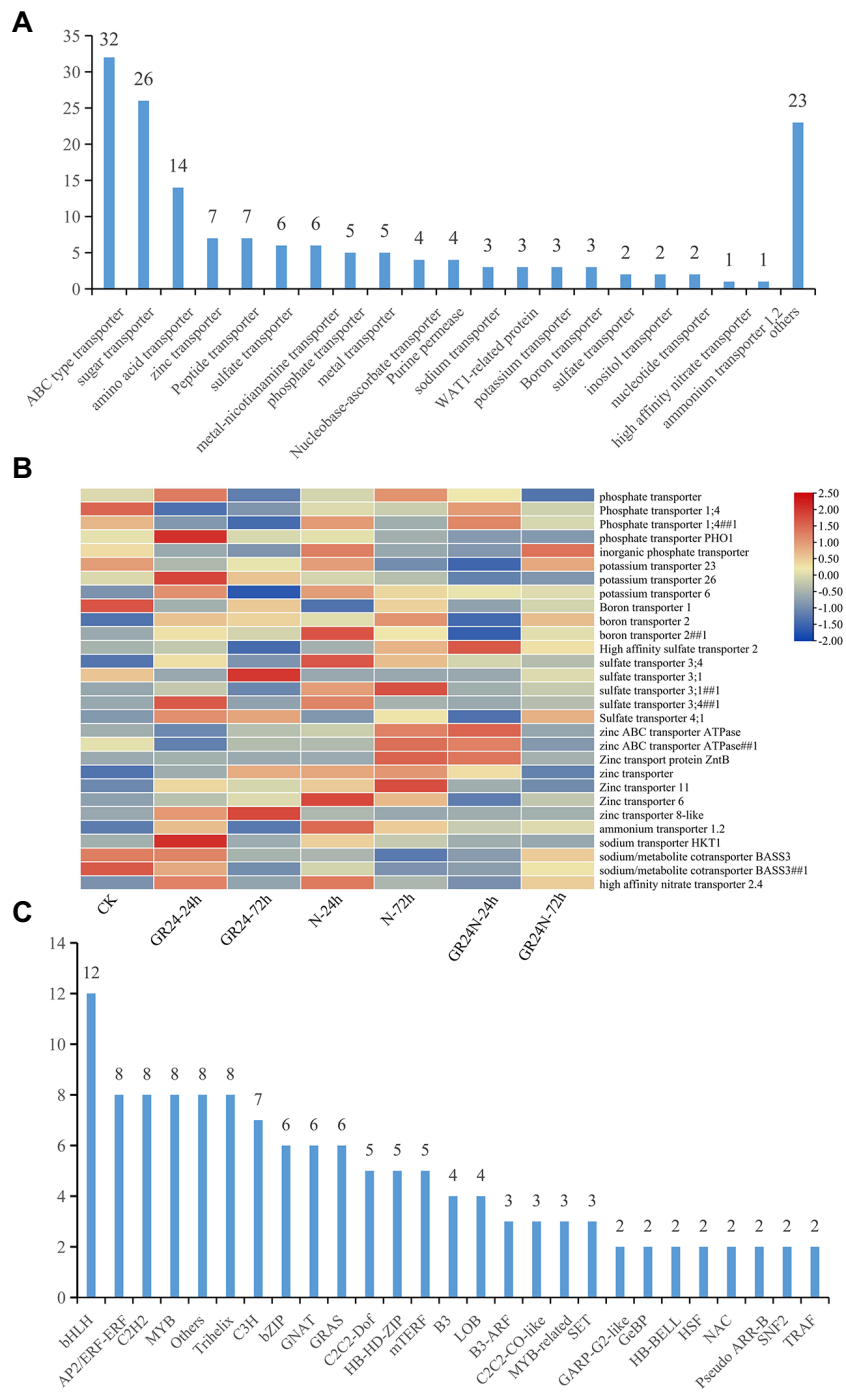
Screening of differentially expressed genes related to transcription factors and substance transporters. **(A)** Identification of differential genes associated with transporters. **(B)** The heat map shows the expression levels of nutrient-related transporters in each treatment. **(C)** Identification of differentially expressed genes that are transcription factors. A total of 26 families of transcription factors were obtained.

**Figure 5:**
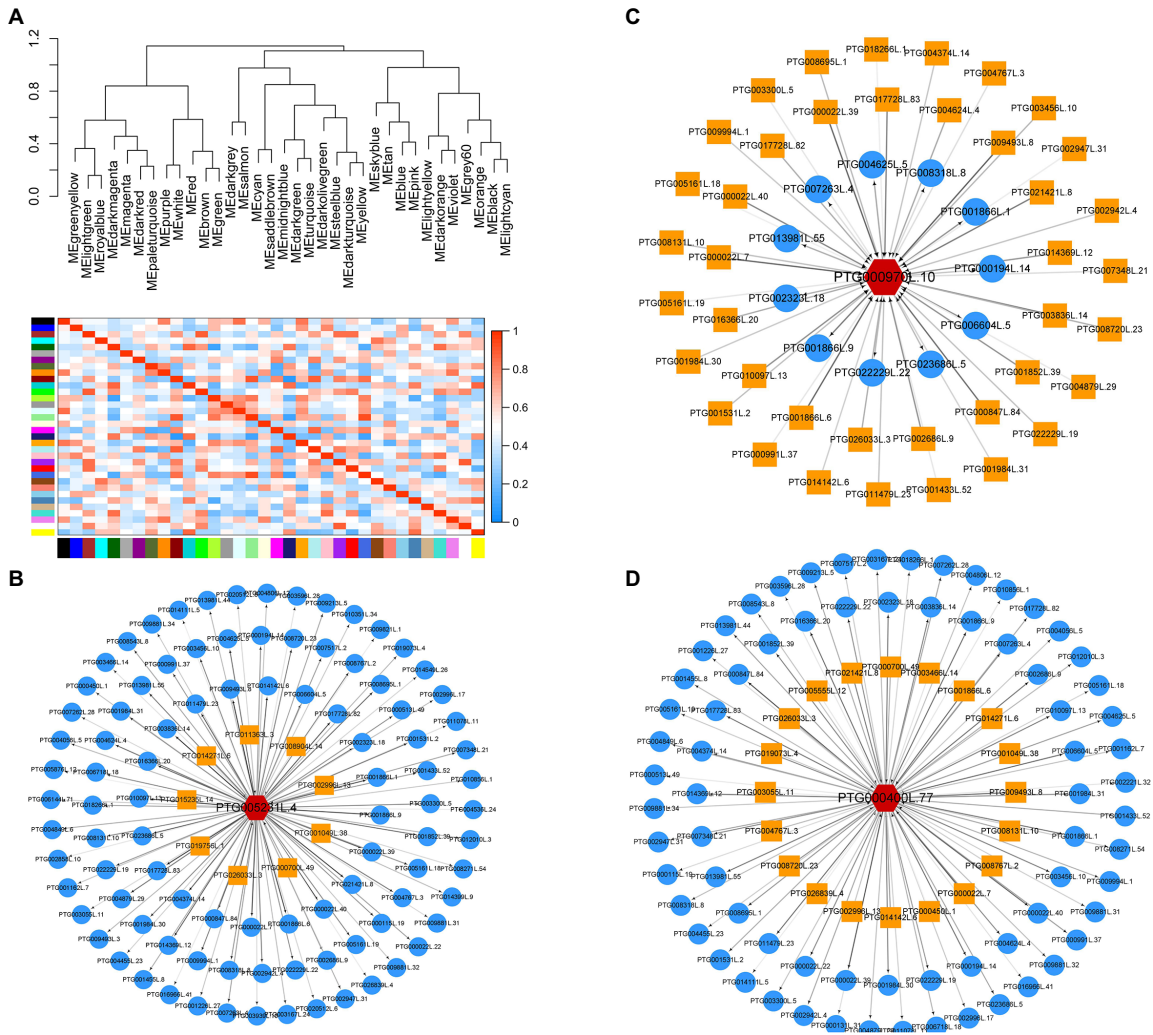
Identification of WGCNA modules associated with nutrient-related transporters under additional N deposition and GR24 treatments. **(A)** Heat map of the correlation between modules. A total of 35 modules were identified. Cytoscape representation of three nutrient-related transporters including Phosphate transporter 1;4 (evm.TU.PTG005231L.4) **(B)**, High affinity sulfate transporter 2(evm.TU.PTG000970L.10) **(C)** zinc ABC transporter ATPase(evm.TU.PTG000400L.77) **(D)**. The yellow box represents the upstream of the target gene, and the blue circle represents the downstream.

Further analysis was conducted to explore DEGs related transporters. A total of 20 families of transporters were identified (Figure 4A). The findings showed that 29 DEGs were transporters of nutritional elements (Figure 4B). The change of expression of these element transporters may be the main reason for the difference of nutrient absorption of *T. grandis* under different treatments. Transcription factors are the regulatory factors that mainly regulate transcription of some genes after cells respond to external stress. Further analysis was conducted on the transcription factors of different genes. The findings showed that the transcription factors may be involved in the signal transduction of N deposition and strigolactone and regulation of the nutrient transport vectors. A total of 26 families of transcription factors were identified (Figure 4C). SLs may regulate the expression of transporters by mediating the expression of these transcription factors. Further, WGCNA analysis shows that all unigenes were divided into 35 modules, which were defined by different color codes (Figure 5). Three unigene associated with the elements transporters were in the “blue” module including a Phosphate transporter 1; 4 (evm.TU.PTG005231L.4), a High affinity sulfate transporter 2(evm.TU.PTG000970L.10), and a zinc ABC transporter ATPase (evm.TU.PTG000400L.77). According to the weight value, we made the network interaction diagram of three genes.

### Effects of Additional N Deposition and GR24 Treatment on Soil Microbial Community Structure

**Figure 6:**
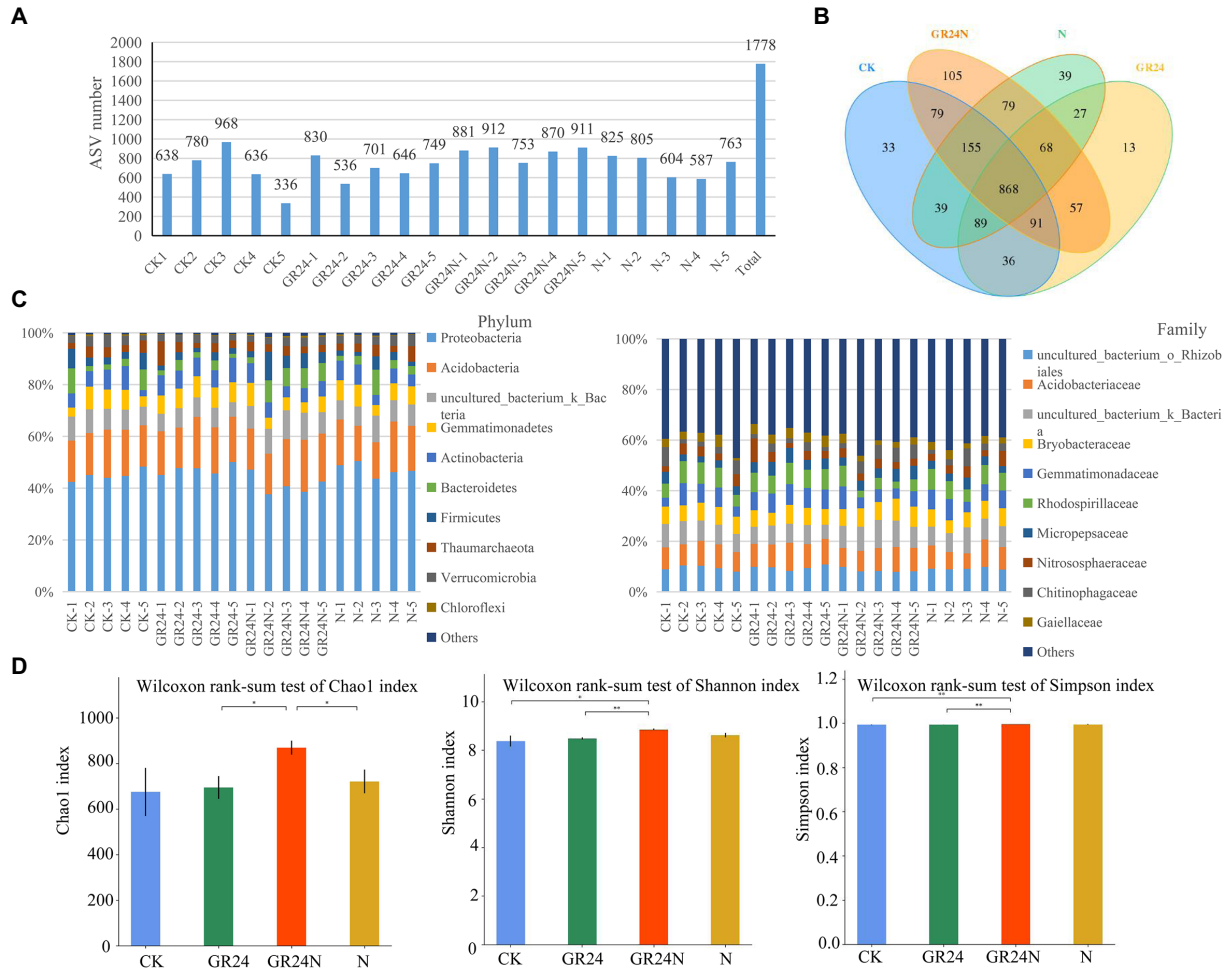
Effects of additional N deposition and GR24 treatments on soil bacteria composition. **(A)** The number of ASVs obtained from each samples. ASVs were obtained by denoising the sequence using the dada2 method included in the QIIME2 software. **(B)** Venn diagram between treatments. **(C)** The histogram shows the species composition of soil bacteria in each sample (relative abundance at phylum and family levels). **(D)** Histogram of group differences in alpha diversity index. The significance of factors and the interaction between factors is marked at the top of the figure, where “*” represents *p* < 0.05 and “**” represents *p* < 0.01.

Previous studies report that nitrogen deposition affects growth, reproduction, and activity of soil microorganisms (Díaz-Álvarez et al., 2018). In addition, nitrogen deposition changes the structure and function of soil microbial community. Therefore, 16S sequencing was conducted to explore the effect of nitrogen deposition on soil microorganisms associated with *T. grandis.* A total of 2,819,899 pairs of PE reads were obtained from 20 samples (*n* = 5; Supplementary Table S4). A total of 1,402,819 clean reads were generated after PE reads quality control and splicing. Each sample resulted in at least 35,711 clean reads, with an average of 70,141 clean reads. A total of 1778 amplicon sequence variants (ASVs) were obtained using the dada2 method in qiime2 software (Figure 6A and Supplementary Table S5). The findings showed that 868 ASVs were common to the four groups of samples (Figure 6B). Additional N deposition and GR24 treatment modulated the structure of soil bacteria at different classification levels (Figure 6C). Alpha diversity analysis of soil bacterial community structure under different treatments showed that the Chao1 and Shannon index were significantly higher in the additional N deposition+GR24 treatment group compared with the control group (Figure 6D). Shannon index and rank abundance curve in the tested soil samples gradually flattened, revealing a reasonable number of sequences (Supplementary Figures S1C,D). PERMANOVA (Adonis) analysis showed significant differences in microbial structure among the different treatments (Supplementary Figure S3).

### Effect of Additional N Deposition and GR24 Treatment on Relative Abundance of Soil Bacteria

**Figure 7:**
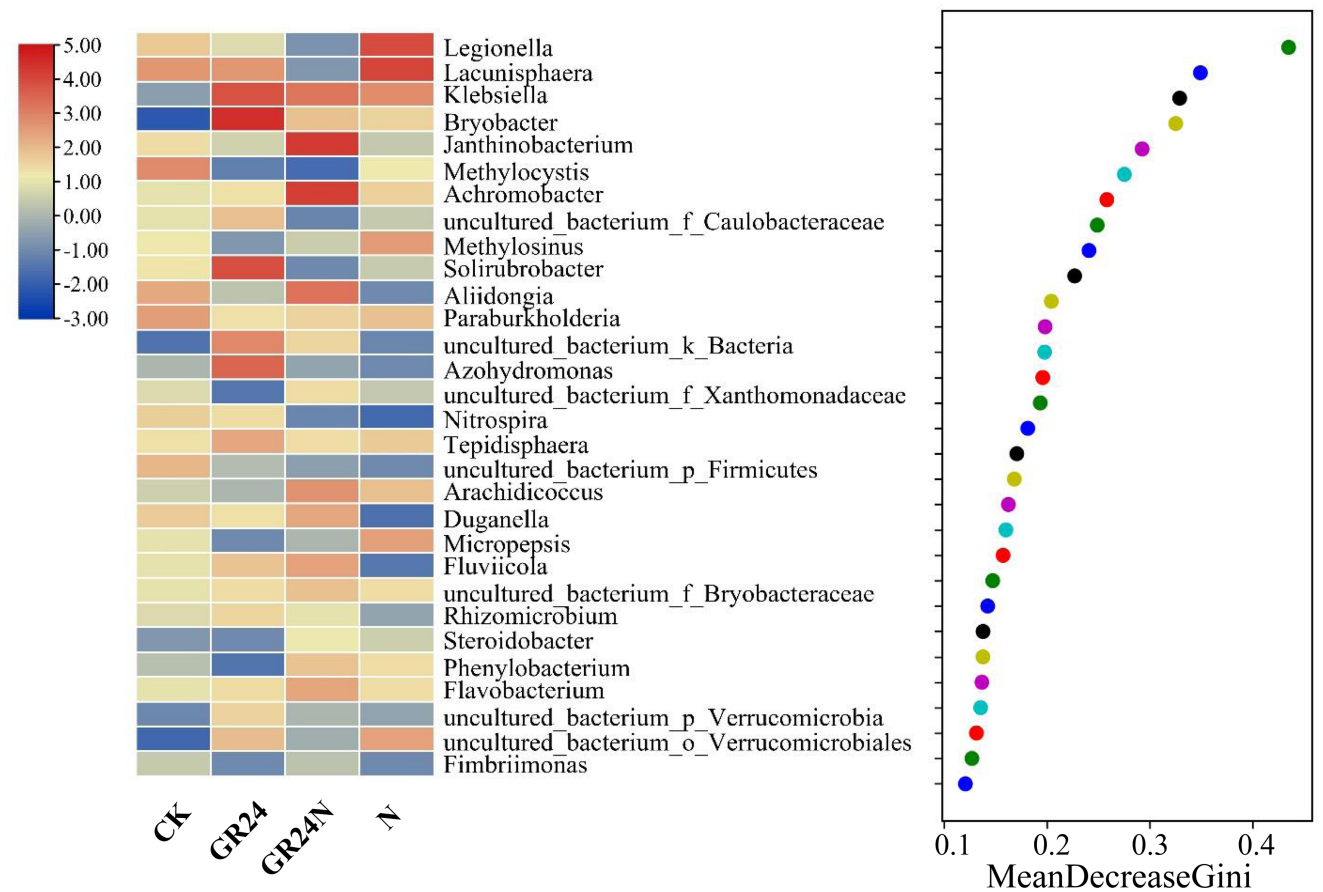
Random forest classification analysis of dominant bacteria in additional N deposition and GR24 treatments.

**Figure 8:**
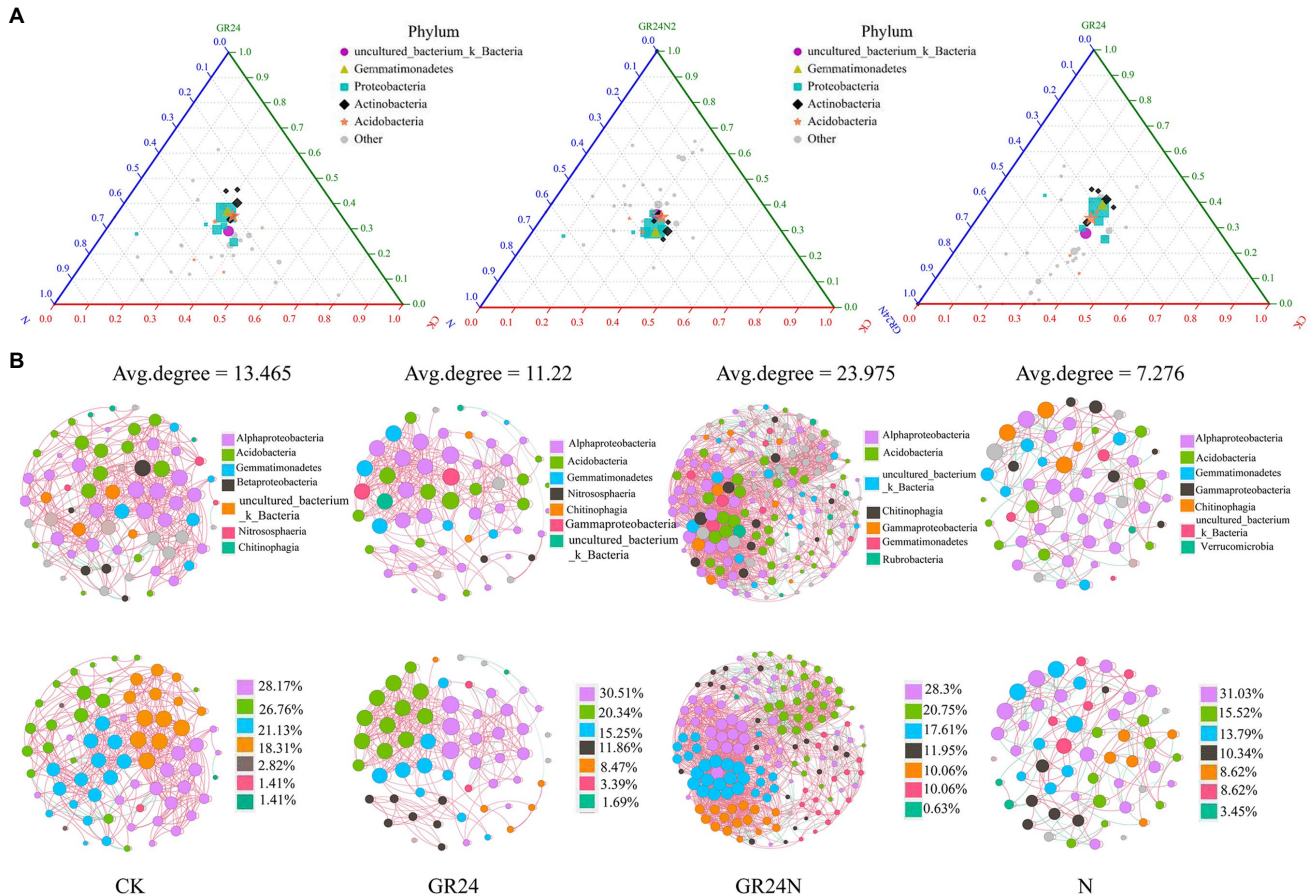
Effects of additional N deposition and GR24 treatments on soil microbiome structures and diversities. **(A)** Ternary phase diagram of mock, additional N deposition and GR24 treatments soil microbiome. The species composition of the three groups of samples is compared and analyzed, and the proportion and relationship of different species in the samples can be visually displayed through the triangular graph. The circle in the triangle represents the species classification of all class levels contained under the phylum level, the size of the circle represents the average relative abundance of species, and the colored circle in the legend represents the species classification of the five phylum levels with the highest abundance. **(B)** Network of soil microbial populations additional N deposition and GR24 treatments at class level. Red lines indicate positive correlations and green lines for negative correlations. Node area is proportional to node degree calculated from each ASV abundance correlation. Correlation coefficients greater than 0.6 and P less than 0.05 were shown in the network.

Random forest classification model was used to evaluate discriminatory taxa in soils under different treatments (Figure 7). The results revealed that Legionella, Lacunisphaera, Klebsiella, Bryobacter, and Janthinobacterium were significantly enriched in the additional N deposition or GR24 treatment soil microbiomes. A ternary diagram was generated which showed that some Proteobacteria were enriched in the additional N deposition-treated soil, whereas some acidobacteria were enriched in the control group soil. Co-occurrence network analysis showed that composition of soil microbial community at the classes level changed significantly under different treatments (Figure 8). The top five most abundant classes in the control group were Alphaproteobacteria, Acidobacteria, Gemmatimonadetes, Betaproteobacteria, and uncultured_bacterium_k_Bacteria. The top five abundant classes in GR24 treatment group were Alphaproteobacteria, Acidobacteria, Gemmatimonadetes, Nitrososphaeria, and Chitinophagia. The top five abundant classes in additional N deposition treatment group were Alphaproteobacteria, Acidobacteria, Gemmatimonadetes, Gammaproteobacteria and Chitinophagia. The top five most abundant classes in additional N deposition +GR24 treatment group were Alphaproteobacteria, Acidobacteria, uncultured_bacterium_k_Bacteria, Chitinophagia, and Gammaproteobacteria.

## Discussion

Nitrogen deposition affect the supply of soil nutrients in the ecosystem (Lü and Han, 2010). Change in soil nutrient content directly affects absorption and utilization of soil nutrients by plants and modulates the stoichiometric characteristics of plants (Tessier and Raynal, 2003). Plants respond to changes in the external environment by activating the signal cascade that modulates the expression pattern of downstream genes. In addition, they change their morphological structure and physiological characteristics to adapt to the new environment (Jiang et al., 2021). Strigolactones, auxin, and cytokinin work synergistically to modulate lateral branch growth of plants and maintain the aboveground phenotypes of plants (Umehara et al., 2008). SLs affect root growth and root hair development, respond to external conditions of plant nitrogen and phosphorus deficiency, and regulate key hormone networks (Villaecija-Aguilar et al., 2019). Studies on different plant species report that nutrient levels, such as N/P in soil, are key regulators of SLs biosynthesis and secretion (Yoneyama et al., 2007; Kohlen et al., 2011). Notably, SLs also play important roles in the process of plant response to stress. SLs deficient mutants are more sensitive to drought and salt stress compared with the wild type (Torres-Vera et al., 2014; Li et al., 2020). The mutant phenotype can be restored by addition of exogenous GR24 (Ha et al., 2014). Wild-type plants treated with GR24 are more resistant to drought and salt stress compared with untreated plants (Ha et al., 2014). In the present study, transcriptome data from *T. grandis* shoots and Bacterial 16S rDNA sequencing data from the soil subjected to various treatments were used to evaluate the relationship between additional N deposition and SLs.

Differential gene analysis is an effective tool to explore gene expression changes over time (Zhan et al., 2018). In the current study, the number of DEGs after 72 h in the three treatment groups varied greatly. The findings showed that 205 and 130 DEGs were upregulated and downregulated, respectively, under the additional N deposition+GR24 treatment for 72 h. This finding shows a relationship between additional N deposition and SLs in *T. grandis* leaves. Nitrogen element and SL are implicated in regulation of plant metabolic pathways. Brassinosteroid biosynthesis pathway was significantly enriched under additional N deposition treatment for 24 h, GR24 treatment for 72 h and additional N deposition+ GR24 treatment for 24 h and 72 h. Brassinosteroids play an important role in plant growth and development and adaptation to the external environment (Ji et al., 2011; Gou et al., 2012). Additional N deposition or GR24 treatment affected synthesis of brassinosteroids. Amino sugar and nucleotide sugar metabolism and Cyanoamino acid metabolism were significantly enriched under additional N deposition treatment and GR24 treatment. These results show that SLs plays an important role in regulating the genes involved in these pathways.

Nitrogen plays a key role in modulating plant growth (Kiba et al., 2018). The content of nitrogen in plants or its effective utilization in soil directly modulates changes in various nutrient elements in plants and regulates the nutrient balance in plants (Li et al., 2012; Ravazzolo et al., 2020). Excessive nitrogen deposition reduces the growth of plants. This is because the excess nitrogen affects nutrient balance in plants (Bobbink and Roelofs, 1998). Nitrogen deposition changes the content of other elements including carbon, nitrogen, and phosphorus, which play important roles in plant physiological processes and may also affect some key processes in the ecosystem (Ruiqiang et al., 2016; Chen et al., 2017). Our results showed that short-term nitrogen deposition treatment induces a decrease in phosphorus content in the roots of *T. grandis*. GR24 treatment promotes increase in P content in leaves of *T. grandis*. The findings showed that the content of S, Fe, Mn, and Zn changed under additional N deposition treatment as well as GR24 treatment. Changes in the content of these elements may be attributed to dysregulation of the expression of the relevant transporters under N deposition or GR24 treatment. Environmental factors, such as soil nutrients, may directly or indirectly regulate the biosynthesis of SLs. Urea and nitrate may contribute to the synthesis and secretion of SLs in red clover root, while phosphorus and ammonium salts might hinder the synthesis (Yoneyama et al., 2001). SLs and its key signaling genes can affect the expression of transcription factors and thus affect plant root growth (Sun et al., 2021; Sylwia et al., 2021). celements are regulated by many transcription factors (Gong et al., 2020). Therefore, the effect of SLs on element content under the background of nitrogen deposition may be realized by regulating the expression of transporter by transcription factors.

High levels of nitrogen deposition leads to soil acidification, imbalance in storage of soil nutrient elements, changes soil microbial community structure, and decreases microbial biomass (Waldrop et al., 2004; Lu et al., 2014). The specific functional microorganisms driving the nitrogen cycle have inconsistent responses to environmental changes, which can easily lead to the weakening of the coupling between nitrogen cycle links under the condition of increased nitrogen deposition, thus breaking the balance of nitrogen cycle (Levy-Booth et al., 2014). When growth is limited by phosphorus, plants will absorb excess N to synthesize phosphatase or induce microorganisms to secrete phosphatase, and the increase of phosphatase activity will release phosphorus absorbed in minerals and organic matter in large quantities, thus increasing the availability of soil P (Treseder and Vitousek, 2001). Phosphatase synthesis requires enough nitrogen (Treseder and Vitousek, 2001). The increase of soil P availability can promote the absorption and utilization of P by N-fixing bacteria and alleviate the restriction of P on nitrogen-fixing microorganisms in tropical and subtropical areas, which is an important factor to ensure the nitrogen fixation function of soil microorganisms under the background of high N deposition (Benjamin et al., 2008). The findings of the present study indicated that short-term nitrogen deposition and SLs treatment changed the composition and structure of soil bacteria, with significant changes observed in the group subjected to additional N deposition + GR24. The *α* diversity in additional N disposition + GR24 group was significantly different compared with that of the other treatment groups. Previous results indicated that nitrogen application had no significant effect on bacterial diversity, however, it significantly changed composition of microbial community (Fierer et al., 2012). The change in microbial diversity can be attributed to increase in nitrogen availability. In the current study, nitrogen deposition treatment increased the relative abundance of gammaproteobacteria and chitinophagia, whereas GR24 treatment increased the relative abundance of nitrososphaeria and chitinophagia. Previous studies report that Proteobacteria comprises a variety of metabolic species and is widely involved in the biochemical cycle of carbon, nitrogen, and other elements in soil (Kersters et al., 2006; Fenliang et al., 2014). Conversion of ammonia to nitrite is a key step in the nitrogen cycle. Microorganisms involved in this process mainly include ammonia-oxidizing bacteria, which are members of betaproteobacteria and gamma Proteobacteria, and ammonia-oxidizing organisms which are members of archaea group (Hatzenpichler et al., 2008; Prosser and Nicol, 2008). Abundance of Gammaproteobacteria significantly increases with increase in contents of nitrogen and phosphorus (Simek et al., 2006). Nitrososphaeria is the most widely distributed Archaea on earth and is involved in ammonia oxidation (Sheridan et al., 2020). Chitinophagia class comprises endosporogenic microorganisms that degrade plant-derived carbohydrates in terrestrial ecosystems (Kishi et al., 2017). The results of this study showed that microorganisms implicated in nitrogen cycle were significantly enriched after GR24 treatment. Random forest classification analysis showed that Legionella was an important differentially expressed flora. The abundance of Legionella increased under nitrogen treatment and decreased under GR24 treatment. Legionella is a pathogenic bacteria (Atlas, 1999) widely distributed in various natural and artificial environments (van Heijnsbergen et al., 2016). The relative abundance of Bryobacter increased under GR24 treatment. Abundance of Bryobacter was positively correlated with soil health and directly correlated with available P concentration (Huang et al., 2020; Liang et al., 2020). These results indicate suggest that SLs promote increase in abundance of beneficial bacteria and reduction of harmful bacteria in soil.

## Conclusion

In the present study, the effects of strigolactones on genetic responses and soil microorganisms associated with *T. grandis* were explored under simulated nitrogen deposition u*s*ing high-throughput sequencing techniques. A total of 4,008 differentially expressed genes were identified in the study. These genes were associated with enrichment of multiple GO terms and metabolic pathways. Nitrogen deposition and GR24 treatment modulated the content of nutrient elements. A total of 1,778 ASVs were generated by 16S sequencing. Random forest classification model revealed that Legionella, Lacunisphaera, Klebsiella, Bryobacter, and Janthinobacterium were significantly enriched in the additional N deposition group or GR24 treatment group. Co-occurrence network analysis showed that species composition of soil microbial community was significantly different under the different treatments. These results indicate an association between N deposition and strigolactone activity.

## Data Availability Statement

The datasets presented in this study can be found in online repositories. The names of the repository/repositories and accession number(s) can be found at: https://www.ncbi.nlm.nih.gov/bioproject/PRJNA815869; https://www.ncbi.nlm.nih.gov/bioproject/PRJNA815930.

## Author Contributions

WY and JW designed the research. CY, QW, SZ, HZ, and WjC did running the experiments and data analysis and statistics. WcC and HL analyzed the data. CY, QW, WY, and JW did the manuscript writing and revising. All authors contributed to the article and approved the submitted manuscript.

## Funding

This work was supported by the National Natural Science Foundation of China (Grant nos. 32171830 and 31800579); the breeding of new varieties of Torreya grandis Program (2021C02066-11); “Pioneer” and “Leading Goose” R&D Program of Zhejiang (2022C02061); and Scientific R&D Foundation for Talent Start-up Project of Zhejiang A&F University (2020FR073).

## Conflict of Interest

The authors declare that the research was conducted in the absence of any commercial or financial relationships that could be construed as a potential conflict of interest.

## Publisher’s Note

All claims expressed in this article are solely those of the authors and do not necessarily represent those of their affiliated organizations, or those of the publisher, the editors and the reviewers. Any product that may be evaluated in this article, or claim that may be made by its manufacturer, is not guaranteed or endorsed by the publisher.

## Footnotes

^1^ https://cutadapt.readthedocs.io/en/stable/

^2^ https://gephi.org/

## Supplementary Material

Supplementary Figure S1 Comparison of the reproducibility of transcriptome and 16S sequencing. Principle component analysis (PCA) for transcriptome(A) and bacterial community 16S rRNA gene sequences(B). Shannon index rarefaction curve(C) and Rank Abundance Curve (D) were used to assess the depth of coverage for each sample.

Supplementary Figure S2 Analysis of the differentially expressed unigenes (DEGs) under N deposition or GR24.

Supplementary Figure S3 Permanova analysis box diagram. Y-axis represents Beta distance; The box above ‘All between ‘represents the Beta distance data of samples between all groups, while the box above ‘All within ‘represents the Beta distance data of samples within all groups. The box below represents the Beta distance data of samples within different groups.

Supplementary Table S1 Sequencing output statistics of 21 RNAseq samples.

Supplementary Table S2 The expression profile of all identified genes.

Supplementary Table S3 Detailed information of identified differentially expressed genes.

Supplementary Table S4 Summary of 16S raw data processing.

Supplementary Table S5 ASV abundance of all the 16S sequencing samples.
