# Supplementary figures and images for: Effects of Strigolactone on Torreya grandis Gene Expression and Soil Microbial Community Structure Under Simulated Nitrogen Deposition
Source: Front Plant Sci. 2022 Jun 2;13:908129. doi: 10.3389/fpls.2022.908129 (PMC9201785; doi:10.3389/fpls.2022.908129)

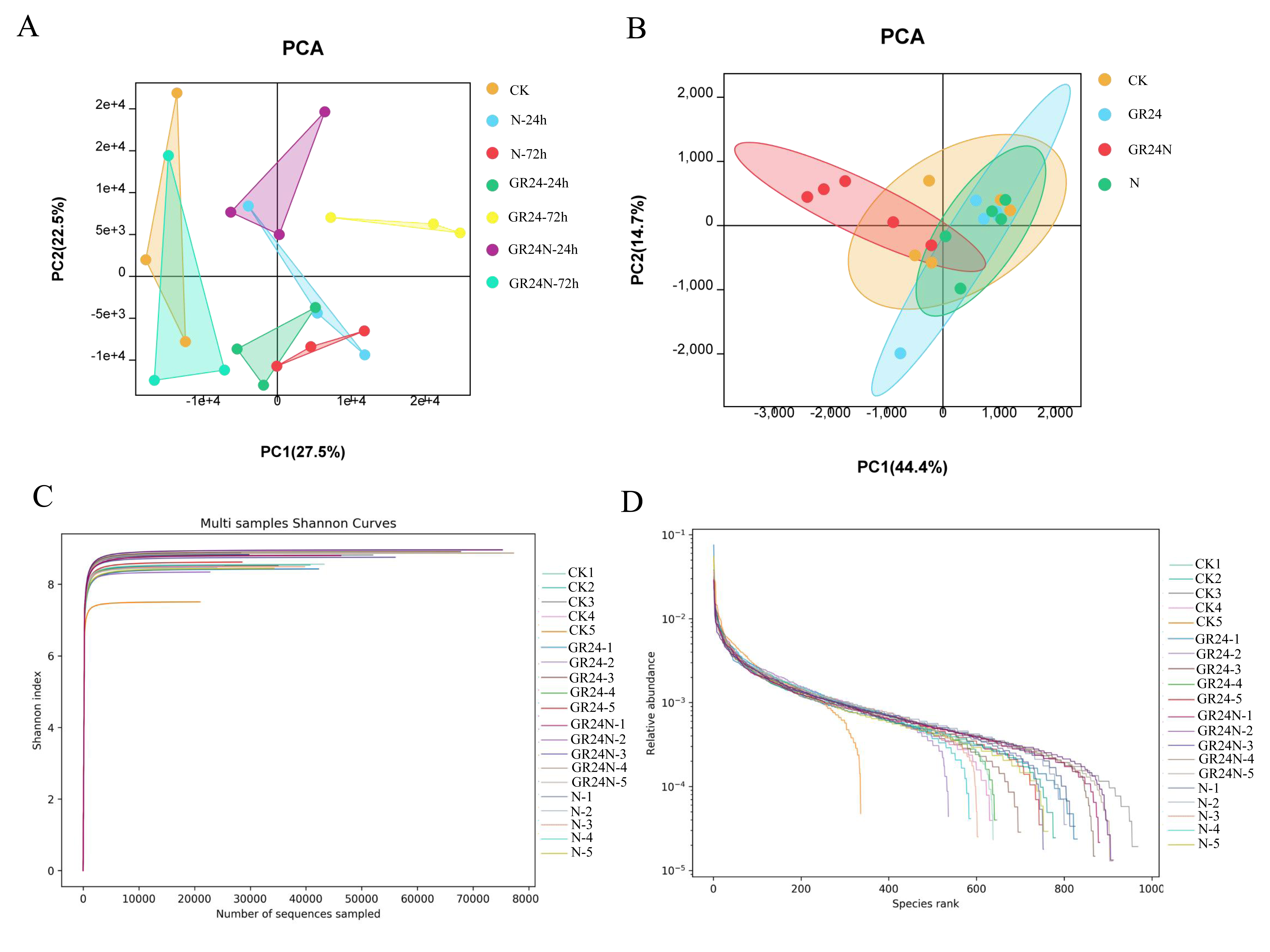

Supplement: Supplementary Figure S1 — Comparison of the reproducibility of transcriptome and 16S sequencing. Principle component analysis (PCA) for transcriptome(A) and bacterial community 16S rRNA gene sequences(B). Shannon index rarefaction curve(C) and Rank Abundance Curve (D) were used to assess the depth of coverage for each sample. [file Image_1.TIF]

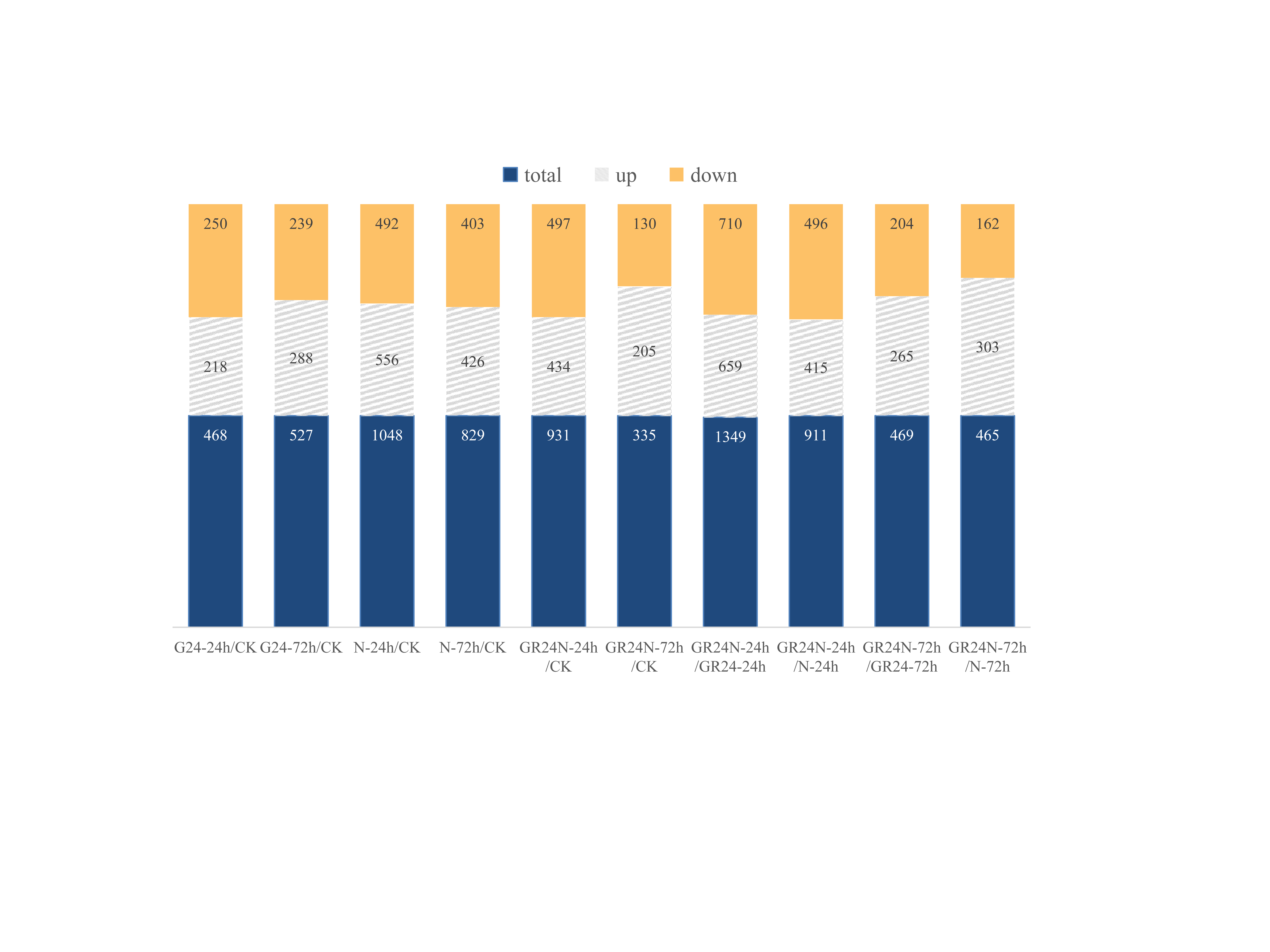

Supplement: Supplementary Figure S2 — Analysis of the differentially expressed unigenes (DEGs) under N deposition or GR24. [file Image_2.TIF]

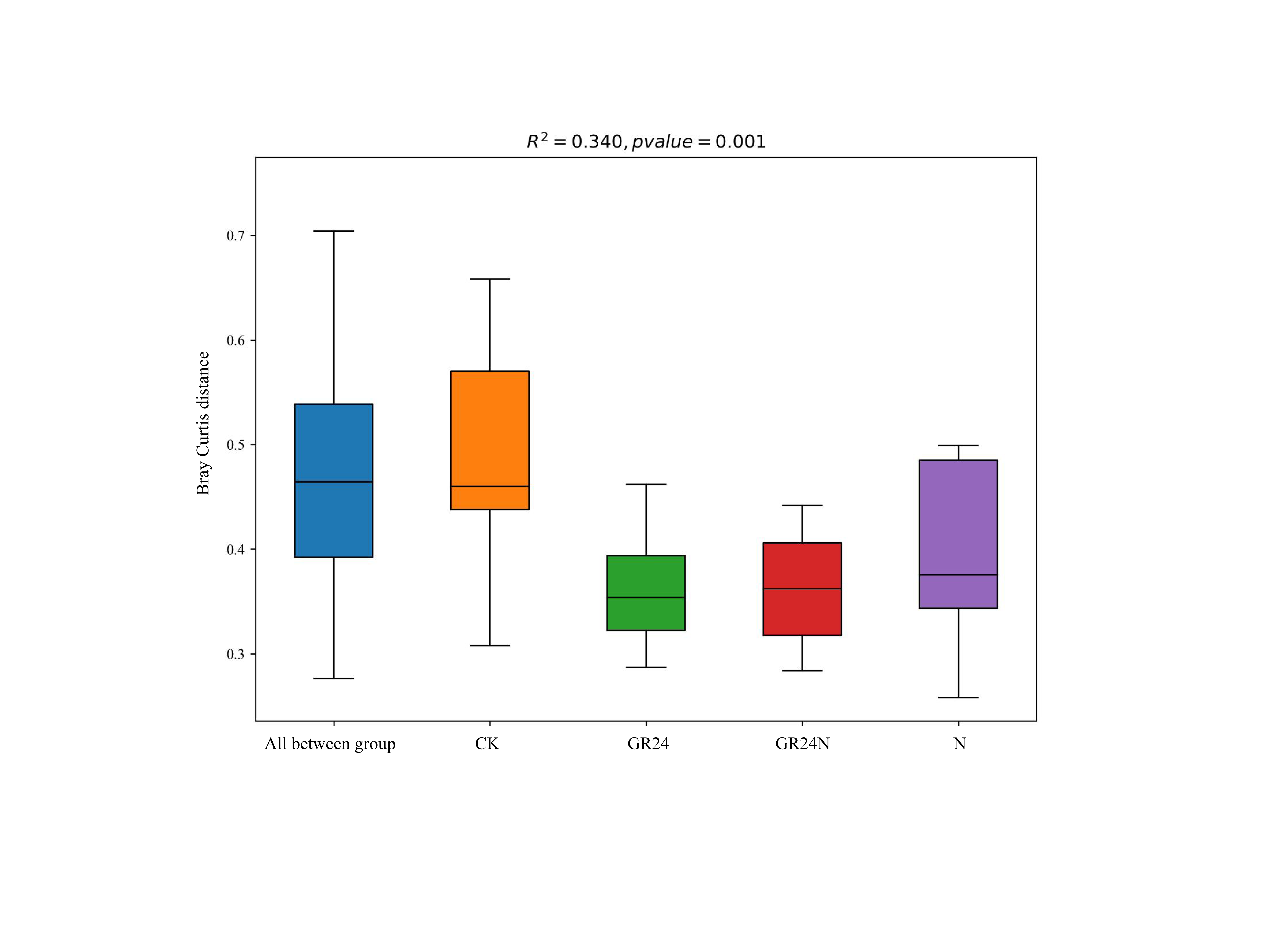

Supplement: Supplementary Figure S3 — Permanova analysis box diagram. Y-axis represents Beta distance; The box above ‘All between ‘represents the Beta distance data of samples between all groups, while the box above ‘All within ‘represents the Beta distance data of samples within all groups. The box below represents the Beta distance data of samples within different groups. [file Image_3.TIF]
